# Colonizing opportunistic pathogens (COPs): The beasts in all of us

**DOI:** 10.1371/journal.ppat.1006369

**Published:** 2017-08-10

**Authors:** Lance B. Price, Bruce A. Hungate, Benjamin J. Koch, Gregg S. Davis, Cindy M. Liu

**Affiliations:** 1 Milken Institute School of Public Health, George Washington University, Washington DC, United States of America; 2 Division of Pathogen Genomics, Translational Genomics Research Institute, Flagstaff, Arizona, United States of America; 3 Center for Ecosystem Science and Society, Northern Arizona University, Flagstaff, Arizona, United States of America; 4 Department of Biological Sciences, Northern Arizona University, Flagstaff, Arizona, United States of America; Geisel School of Medicine at Dartmouth, UNITED STATES

## Introduction

Colonizing opportunistic pathogens (COPs) are microbes that asymptomatically colonize the human body and, when the conditions are right, can cause infections. Their ability to persist indefinitely and to be transmitted without detection [1] gives COPs a unique epidemiology that warrants special consideration. There are examples of COPs among bacteria, fungi (e.g., *Candida albicans* [2]), protozoa (e.g., *Blastocystis* [3, 4]), and viruses (e.g., *Rhinovirus* [5]), but bacterial COPs are of particular relevance because of their major contribution to today’s antibiotic resistance crisis. The COPs include a long list of notorious bacteria that live double lives as passive stowaways and virulent foes. Some of the best-known COPs include *Staphylococcus aureus*, extraintestinal pathogenic *Escherichia coli* (ExPEC), *Klebsiella pneumoniae*, and *Streptococcus pneumoniae* (Table 1). Their capacity for benign coexistence with humans belies their alter egos that exact a heavy burden of human disease. For example, in the United States, ExPEC bloodstream infections kill as many as 40,000 people annually [6], but, ExPEC are also benign colonizers in the gastrointestinal tract [7]. Host factors, including age, sex, health status, anatomy, and behavior, all play profound roles in infection susceptibility and severity [8–10]. In particular, immunocompromised individuals are at excess risk for infections caused by diverse bacteria, including COPs [11, 12] and even commensals. Yet, health status is not the sole determinant of infection by COPs. For example, healthy women more frequently suffer from urinary tract infections than men because of anatomical differences, including shorter urethrae. Likewise, healthy children more commonly suffer from acute otitis media than adults due to their shorter, flatter eustachian tubes [13].

**Table 1:** Common bacterial colonizing opportunistic pathogens, simple opportunistic pathogens, and frank pathogens.

COPs	Reservoir/Site of Colonization	Associated Human Diseases
*Staphylococcus aureus*	Nasal cavity, skin	Cellulitis, abscesses, osteomyelitis, endocarditis, sepsis
*Streptococcus pneumoniae*	Nasopharynx	Otitis media, pneumonia, sepsis
ExPEC	Oral cavity, skin, intestinal tract	Cystitis, pyelonephritis, meningitis, sepsis
*Klebsiella pneumoniae*	Oral cavity, skin, intestinal tract	Cystitis, pneumonia, sepsis
**SOPs**		
*Vibrio vulnificus*	Raw/undercooked seafood, warm costal water	Wound infection, hemorrhagic bullae, sepsis
*Mycobacterium marinum*	Contaminated water sources (e.g., untreated pools, fish aquaria)	Granuloma, tenosynovitis, osteomyelitis
*Legionella pneumophila*	Freshwaters, contaminated human water systems (e.g., showers, faucets, cooling towers)	Legionnaires’ disease, Pontiac fever
**Frank pathogens**		
*Escherichia coli* 0157:H7	Food animals, food products	Bloody diarrhea, hemolytic uremic syndrome
*Campylobacter jejuni*	Food animals, food products	Watery diarrhea, Guillain-Barre syndrome
*Salmonella enterica* (including *S*. *typhi*)	Food animals, food products	Gastroenteritis, cystitis, typhoid fever, sepsis
*Mycobacterium tuberculosis*	Lungs of infected patients	Tuberculosis

COPs, colonizing opportunistic pathogens; ExPEC, extraintestinal pathogenic *Escherichia coli*; SOPs, simple opportunistic pathogens.

Here, we focus on the ecological features that distinguish COPs from other bacterial pathogens. We explore the public health implications that arise from their unique biology and discuss the research needed to illuminate the dynamics of COPs among their human, animal, and environmental reservoirs. And finally, we consider the commonalities among COPs that may require active monitoring and management. Our examination complements and extends the damage-response framework of pathogenesis proposed by Casadevall and Pirofski [14] without focusing on the pathogenicity or virulence potential of COPs, exploring specifically the host immune response, or delving into mechanisms of how COPs transition from colonization to infection.

## COPs are a distinct subgroup of the opportunistic pathogens

**Fig 1:**
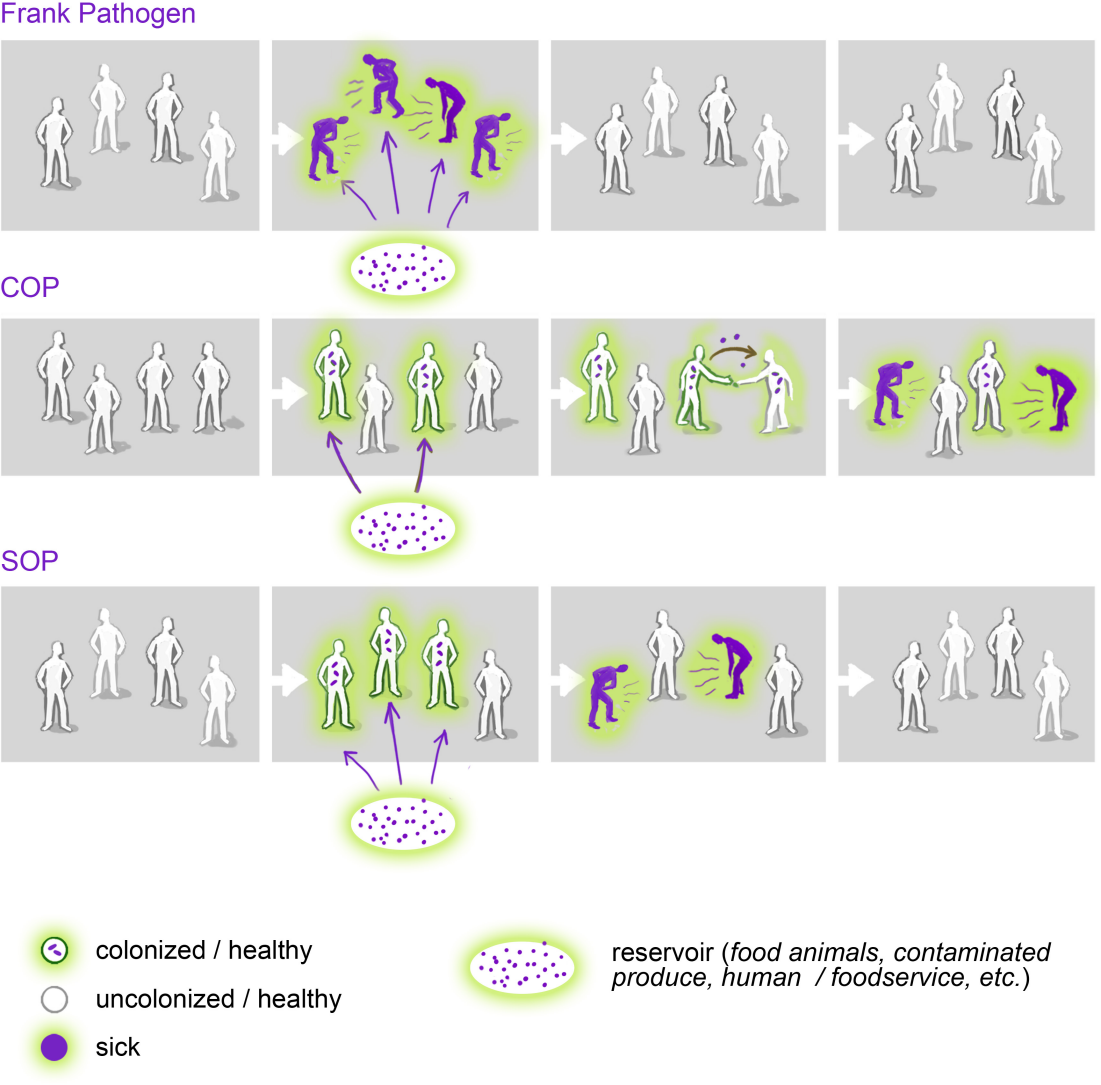
Colonizing opportunistic pathogens (COPs) can persist asymptomatically and indefinitely within a host and may spread silently within the community. These unique features of COPs result in epidemiological patterns distinct from those of frank pathogens and simple opportunistic pathogens (SOPs)—patterns that may have substantial public health consequences—such as the spread of antibiotic resistance. A deeper understanding of COP ecology is needed to reveal the reservoirs and transmission pathways of COPs and to design surveillance programs capable of detecting the otherwise invisible epidemics caused by COPs. (Illustration by Victor Leshyk.)

The broad category of opportunistic pathogens can be divided into 2 distinct subgroups: the COPs and the noncolonizing, simple opportunistic pathogens (SOPs; Fig 1). The defining feature of all opportunistic pathogens is their capacity to cause disease when they are introduced into a susceptible body site or when hosts are immunologically compromised. The reservoirs of opportunistic pathogens are diverse and include food, water, soil, animals, and people with active infections. Whereas SOPs, such as *Vibrio vulnificus*, *Mycobacterium marinum*, and *Legionella pneumophila*, are only present in environmental reservoirs, COPs can also take up long-term residence in or on the human body as part of the “normal” human microbiome. For example, *S*. *aureus* can colonize the human nose asymptomatically and transmit from person-to-person without causing disease [15, 16]. Although COP colonization itself is asymptomatic, it can be an important risk factor for subsequent disease [17]. For *S*. *aureus*, higher-abundance colonization that is detectable by culture is further linked to higher risk of subsequent infection than lower-abundance colonization that is frequently undetected by culture [18].

COPs differ from frank pathogens (Table 1), which can cause acute, chronic, or latent infections that can be symptomatic or asymptomatic [19]. While the latent and reactivation phases of frank pathogens, such as *Mycobacterium tuberculosis* and *Salmonella enterica* serovar Typhi, may mimic the colonization and infection phases of COPs, they are distinct in that these latent infections are typically preceded by an acute infection and maintenance of the latent phase requires an active adaptive immune response [20]. This is distinct from the fully asymptomatic colonization by COPs (Fig 1). The detection of frank pathogens is often associated with a diseased state, whether active or latent, and identifying cases of active or recent infections is usually enough to trace transmission routes. The well-characterized incubation periods of frank pathogens are consistent features of their epidemiology and aid in tracking infections [21]. In contrast, COPs lack predictable periods between colonization and infection, making their epidemiology cryptic. Because of this, COPs can cause insidious epidemics, where new clones transmit widely—even globally—among healthy populations before being recognized. For example, by the time ExPEC strain ST131 was discovered in 2008, it had already made its way to at least 3 continents [22].

## COPs present a special challenge with respect to antibiotic resistance

Today, some of the most important multidrug-resistant bacterial pathogens—methicillin-resistant *S*. *aureus* (MRSA) and carbapenem-resistant Enterobacteriaceae—are COPs. Administration of an antibiotic exerts selective pressure on all bacterial populations within a host. As a result, antibiotics can select for antibiotic-resistant COPs, in addition to commensals within the host microbiome, regardless of whether or not the specific COP was the intended target of the antibiotic treatment. For example, 30% to 50% of healthy adults are carriers of *S*. *aureus* [17, 23, 24], and if a carrier takes a course of cephalosporins for an unrelated infection, the antibiotic is applying collateral selective pressure on the carrier’s *S*. *aureus* population, conferring advantage to resistant subpopulations, including MRSA. Resistant strains that surge under such selective pressure may persist long after the target infection is cleared; this is consistent with the finding that previous antibiotic use significantly increases the risk for future antibiotic-resistant infections [25–29]. Vaccination can also exert selective pressure on target species population, such as in the example of pneumococcal vaccine [30], in which the resultant serotype replacement has shifted the resistance profile seen among invasive *S*. *pneumoniae* infections [31].

## Characterizing the ecology of COPs is paramount to understanding their epidemiology

Characterizing the ecology of COPs will require a new research framework beyond studies of virulence, antibiotic resistance, and epidemiology of individual species. Fully capturing the ecology of COPs will require studying their distribution and dynamics which span 17 orders of spatial magnitude—from the human microbiome (1x10^-5^ m) to global travel and commerce (5x10^11^m). This will require investigating the intra- and inter-species relationships that determine the success and duration of COP colonization, and also the roles of reservoirs (e.g., humans, animals, soil, water, air, and others) in the maintenance and transmission of COPs. Vegetable crops, meat products, water resources, and air can all potentially be contaminated by antibiotic-resistant COPs from food animals and animal wastes [32, 33], in which airborne exposure likely drives the link between proximity of residence to manure application and livestock operations to increased risk of community-acquired MRSA infections [34]. Such ambitious ecological studies may require species- and even strain- or clonal-level focus, but the collective lessons learned can inform how we monitor, forecast, and respond to COPs in general.

The most urgent knowledge gaps related to COPs are the determinants of their colonization after exposure, the duration of colonization, and the transition from colonization to infection. Carefully designed observational studies could provide the samples necessary to close these knowledge gaps, given that empirical studies on human subjects may face ethical challenges, and that animal models—unless they are truly humanized in terms of immunity and resident microbiome—are likely to offer limited insight. Designing informative monitoring and observational studies will require creative and careful planning, as these knowledge gaps are well beyond the scope of typical studies conducted to date. One possible design encompassing the broad scale of COP ecology would be to monitor individuals from regions with a low prevalence of specific multidrug-resistant COPs that travel to regions where they are endemic [35]. One could then assess the microbiome and other host and microbial determinants of colonization and subsequent follow-up could provide insight into the transition from colonization to infection and human-to-human transmissions.

## COPs require new active and integrative surveillance programs

Enhanced surveillance of COPs can enable public health agencies to identify and control emerging COP clones more quickly than is currently possible. Because of the insidious nature of COP epidemics, active surveillance programs that monitor both COPs circulating among asymptomatic carriers in the community and COPs causing clinical infections are crucial. This work will require the development of new molecular methods and could be integrated into existing surveillance programs, such as the National Healthcare and Safety Network, FoodNet, PulseNet, and the National Antimicrobial Resistance Monitoring System [36–39]. Active surveillance of COPs can involve sentinel communities, households, or individuals who are recruited to provide samples from body sites where COPs reside, such as nasal swabs and stool specimens for culture-based and molecular analyses. Current surveillance programs could expand their monitoring to encompass COP clones in clinical infections and in environmental reservoirs. Linking these data could allow an early warning system on emerging COP clones and provide valuable information for tracing community- versus healthcare-associated COP clones. Incorporating whole-genome DNA sequencing can further resolve subtleties ranging from shifts in COP clonal populations to the loss and gain of resistance-encoding mobile genetic elements [40–42].

Both frank pathogens and COPs can cause foodborne zoonotic infections. Infections caused by frank pathogens, such as *Salmonella enterica*, *Campylobacter jejuni*, and *E*. *coli* 0157:H7 (Table 1), can quickly be recognized and controlled by tracing clusters of infections to a common source [43–45]. Foodborne COPs, such as *K*. *pneumonia*e and multidrug-resistant ExPEC, are known to contaminate meat products and can cause infections in consumers [46]; however, because they may persist indefinitely in exposed hosts before causing an infection, disease clusters can be dispersed in time and space, obscuring the source of the pathogen. In addition to the prolonged colonization period, once a foodborne COP successfully colonizes a person, it may also be transmitted person-to-person [47], spreading through the human population to a far greater extent than frank foodborne pathogens. All of this contributes to the challenge of estimating the disease burden of foodborne COPs.

A key target in active surveillance of COPs is the environmental reservoirs (e.g., air, food, and water) and nonhuman “co-host” species such as wild animals, companion animals, and livestock. The widespread use of antimicrobials in livestock [48] makes food animals an important target in monitoring novel resistance elements in COPs and emerging antibiotic-resistant COP clones—this is supported by the recent report of a mobile colistin-resistance element, *mcr-1*, among livestock in China [49]. We will need to monitor COPs throughout the livestock production system—from breeder farms to slaughter—to identify the origins and potential control points for emerging COPs from livestock. Other livestock products, such as wastes and meat, should be integrated into the active surveillance of COPs. Ideally, the active surveillance of environmental reservoirs should be coordinated with the sentinel community-based sites to maximize the integration across scales.

## Concluding thoughts

“Sometimes, it tries to kid me that it's just a teddy bear

or even somehow managed to vanish in the air

and that is when I must beware

of the beast in me.”

*The Beast in Me* by Nick Lowe

COPs are an important subgroup of the opportunistic pathogens that deserve special attention and require novel intervention strategies to curb their heavy public health burden. Understanding both the ecology and the epidemiology of COPs is necessary to identify effective interventions. Antimicrobial therapy is the current mainstay of treating COP infections, but the emergence of broadly multidrug-resistant COPs challenges this modality. The post-antibiotic era requires new ways to prevent COP infections by disrupting colonization rather than trying to treat pan-resistant infections. While much research will have to be conducted on the species or strain level, characterizing the full scope of COP ecology—from persistence in environmental reservoirs to transmission among humans—is key for taming these beasts in all of us.
